# Enhanced cercosporin production by co-culturing *Cercospora* sp. JNU001 with leaf-spot-disease-related endophytic bacteria

**DOI:** 10.1186/s12934-021-01587-2

**Published:** 2021-05-15

**Authors:** Tingan Zhou, Shiyu Yu, Yifan Hu, Yan Zhang, Yuechen Song, Jieyu Chu, Changmei Liu, Yijian Rao

**Affiliations:** 1 Key Laboratory of Carbohydrate Chemistry and Biotechnology, Ministry of Education, School of Biotechnology, Jiangnan University, Wuxi, 214122 People’s Republic of China; 2 School of Pharmaceutical Science, Jiangnan University, Wuxi, 214122 People’s Republic of China

**Keywords:** Cercosporin, Co-culture, Endophytic bacteria, Secretion, Microbial fermentation

## Abstract

**Background:**

Owing to the excellent properties of photosensitization, cercosporin, one of naturally occurring perylenequinonoid pigments, has been widely used in photodynamic therapy, or as an antimicrobial agent and an organophotocatalyst. However, because of low efficiency of total chemical synthesis and low yield of current microbial fermentation, the limited production restricts its broad applications. Thus, the strategies to improve the production of cercosporin were highly desired. Besides traditional optimization methods, here we screened leaf-spot-disease-related endophytic bacteria to co-culture with our previous identified *Cercospora* sp. JNU001 to increase cercosporin production.

**Results:**

*Bacillus velezensis* B04 and *Lysinibacillus* sp. B15 isolated from leaves with leaf spot diseases were found to facilitate cercosporin secretion into the broth and then enhance the production of cercosporin. After 4 days of co-culture, *Bacillus velezensis* B04 allowed to increase the production of cercosporin from 128.2 mg/L to 984.4 mg/L, which was 7.68-fold of the previously reported one. *Lysinibacillus* sp. B15 could also enhance the production of cercosporin with a yield of 626.3 mg/L, which was 4.89-fold higher than the starting condition. More importantly, we found that bacteria B04 and B15 employed two different mechanisms to improve the production of cercosporin, in which B04 facilitated cercosporin secretion into the broth by loosening and damaging the hyphae surface of *Cercospora sp.* JNU001 while B15 could adsorb cercosporin to improve its secretion.

**Conclusions:**

We here established a novel and effective co-culture method to improve the production of cercosporin by increasing its secretion ability from *Cercospora sp.* JNU001, allowing to develop more potential applications of cercosporin.

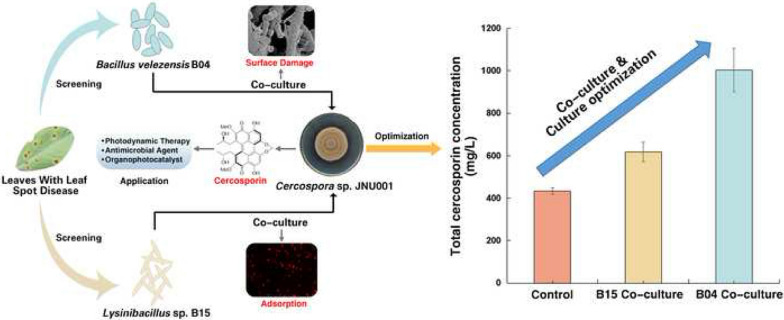

**Supplementary Information:**

The online version contains supplementary material available at 10.1186/s12934-021-01587-2.

## Background

**Fig. 1:**
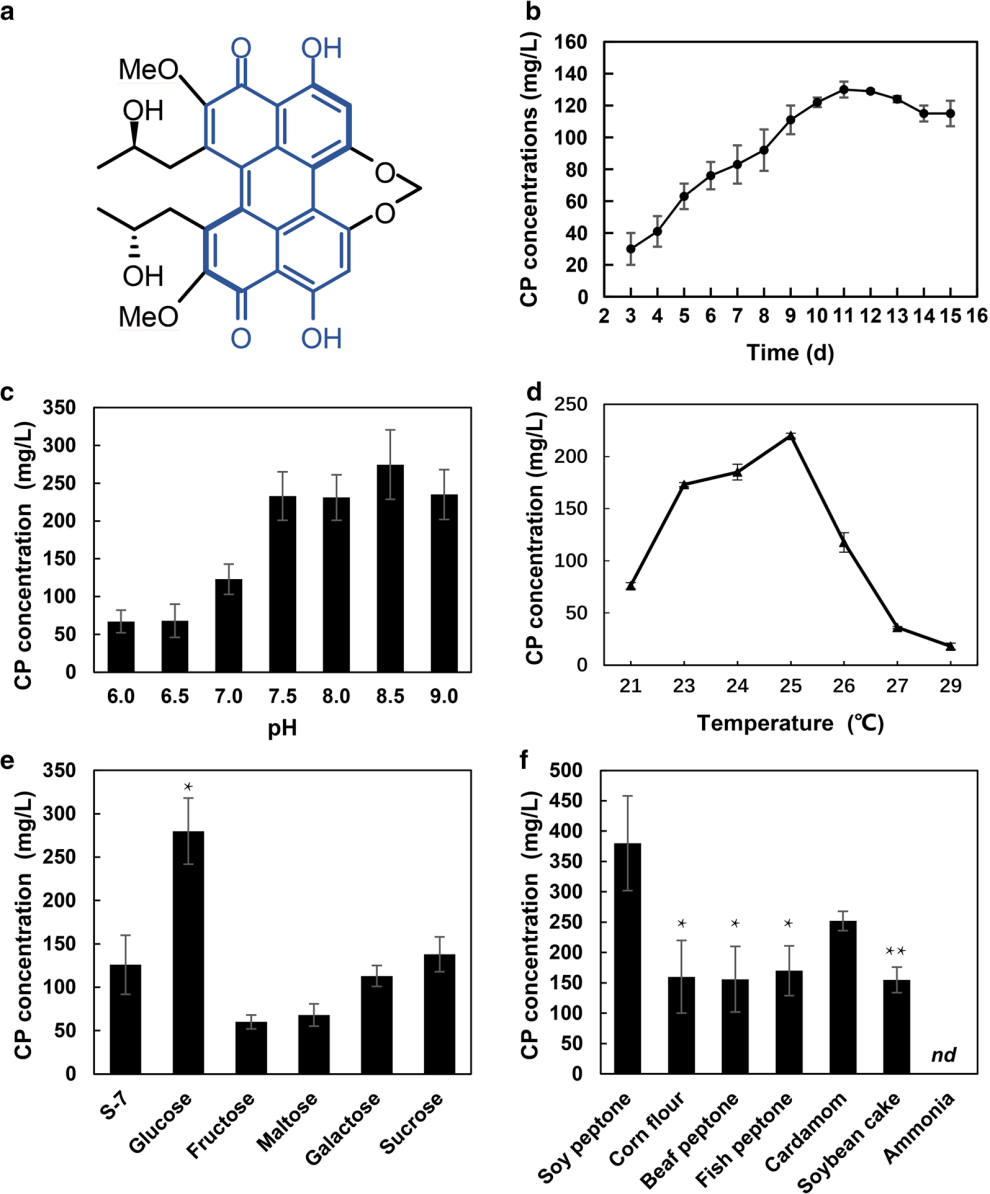
Improvement of CP production by optimizing culture medium and culture conditions. **a** Molecular structure of CP. The characterized 3,10-dihydroxy-4,9-perylenequinone chromophore core structure was labelled in blue. **b** Influence of culture time on CP production. d = day. **c** pH optimization of S-7 medium. **d** Optimization of culture temperature. **e** Selection of carbon source in S-7 medium. **f** Screening of nitrogen source in S-7 medium (cardamom: cardamom powder, ammonia: ammonia sulfate, *nd*: no detected). ^∗^p < 0.05 and ^∗∗^p < 0.01 versus control (carbon/nitrogen source in traditional S-7 medium)

Cercosporin (CP), one of naturally occurring perylenequinonoid pigments (PQPs) with a characterized 3,10-dihydroxy-4,9-perylenequinone chromophore core structure (Fig. 1a), was first isolated in the mycelium of *Cercospora kikuchii* in 1957 and then was widely found in many pathogenic fungus *Cercospora* [1–3], which is a causal agent of leaf spot diseases (LSD) in a wide range of crops [4], including soybean [5], maize [6], and olive [7]. Owing to its excellent properties of photosensitization, it is widely investigated in the aspects of photophysics, photochemistry and photobiology [8–13], and has been used in photodynamic therapy and photophysical diagnosis, or as antimicrobial agents [14–16]. For instance, it has broad applications in the treatment of refractory skin diseases caused by certain fungi and cancer [16, 17]. CP is also a potent inhibitor of protein kinase C (PKC) [18], which regulates numerous intracellular signal transduction, including cell differentiation, cell proliferation and inflammatory response, by controlling the function of other proteins through phosphorylation. More recently, we have developed CP as a new class of metal-free photocatalyst to catalyze a series of chemical transformations, including selective photo-oxidation, C–H activation, C–N coupling and C–S coupling [19–23]. Meanwhile, it has also been utilized to fabricate a novel HARCP/HAp photocatalyst (HexaAcetyl-Reductive Cercosporin/Hydroxyapatite) after a simple structural modification, allowing to efficiently photoremove tetracycline in water pollution under natural sunlight [24]. Based on these applications of CP and potential industrial demands, a large quantity of CP is highly desired.

Currently, CP is mainly produced by total chemical synthesis and microbial fermentation. However, owing to its structural complexity (Fig. 1a), the total chemical synthesis of PQPs, including CP, needs more than twenty steps [18], which limits its practical application. Thus, the production of CP is mainly dependent on microbial fermentation by culturing fungus *Cercospora* sp. [22, 25–27]. So far, many previous studies have focused on increasing the productivity of CP by optimizing numerous fermentation factors, including medium, salts, buffers and ions, and delivered the maximum production of 75.59 mg/L after 31 days culture of a *Cercospora* strain from symptomatic leaves of water hyacinth [26]. However, both of solid-state and liquid fermentation are currently not able to produce stable and high-yield of CP in a large scale within a reasonable culture time. Additionally, its productivity highly varies from different sources of *Cercospora* sp. [2]. Thus, the improvement of CP production through different strategies by a promising *Cercospora* strain is still awaiting to be explored.

Recently, we identified a new CP-producing strain *Cercospora* sp. JNU001, which was isolated from bark of *Taxus chinensis*, with the ability to produce CP at the yield of 128.2 mg/L when it was cultivated under continuous light illumination with S-7 medium after 11 days [22], which is substantially higher than other previous studies, even within a shortened culture time [26]. Therefore, herein we attempted to improve the production of CP using *Cercospora* sp. JNU001 strain. We initiated the improvement of CP production of *Cercospora* sp. JNU001 by typical medium optimization and culture condition optimization, and then employed the co-cultivation method, a powerful ecologically driven approach to increase the production of specific metabolites or to produce some new substances by mimicking natural situations [28–33]. To our delight, besides the enhanced production of CP by typical medium optimization and culture condition optimization, the production of CP could be further improved by co-culturing with two new identified LSD endophytic bacteria *Bacillus velezensis* B04 and *Lysinibacillus* sp. B15. The yields of CP were 984.4 mg/L (*Bacillus velezensis* B04) and 626.3 mg/L (*Lysinibacillus* sp. B15), which were 7.68-fold and 4.89-fold higher than the starting condition, respectively. Furthermore, we found that these two bacteria applied different mechanisms to improve the production of CP.

## Results and discussion

### Determination and optimization of liquid fermentation conditions

Considering the limited production of CP on PDA plate [22], S-7 culture medium was firstly chosen as the basic medium to optimize the liquid fermentation conditions [19, 22, 34], including culture time, medium pH, temperature, carbon source and nitrogen source (Fig. 1b–f). It was found that the production of CP was dramatically increased from 128.2 mg/L to 467.8 mg/L when *Cercospora* sp. JNU001 was cultured at 25 °C with the modified S-7 medium (initial pH = 8.5) (Fig. 1b–d), in which glucose was used as carbon source and soy peptone as nitrogen source (Fig. 1e, f), for 11 days under continuous light illumination. The total amount of CP was 6.19-fold and 3.65-fold higher than the previously reported condition [26], and our original condition [35], respectively. For the *Cercospora* sp. JNU001 strain, its production ability reached the maximum at 11 days and then part of CP was degraded when the culture time was increased (Fig. 1b), which is consistent with previous studies [35]. Surprisingly, the production of CP was almost inhibited when the culture temperature was set at more than 27 °C (Fig. 1d). Moreover, no CP was produced when inorganic ammonia was used as nitrogen source (Fig. 1f). Thus, we obtained the highest productivity of CP after typical optimizations through liquid fermentation, allowing us to further improve CP production by co-culture strategy.

### Screening of leaf-spot-disease-related bacteria

**Fig. 2:**
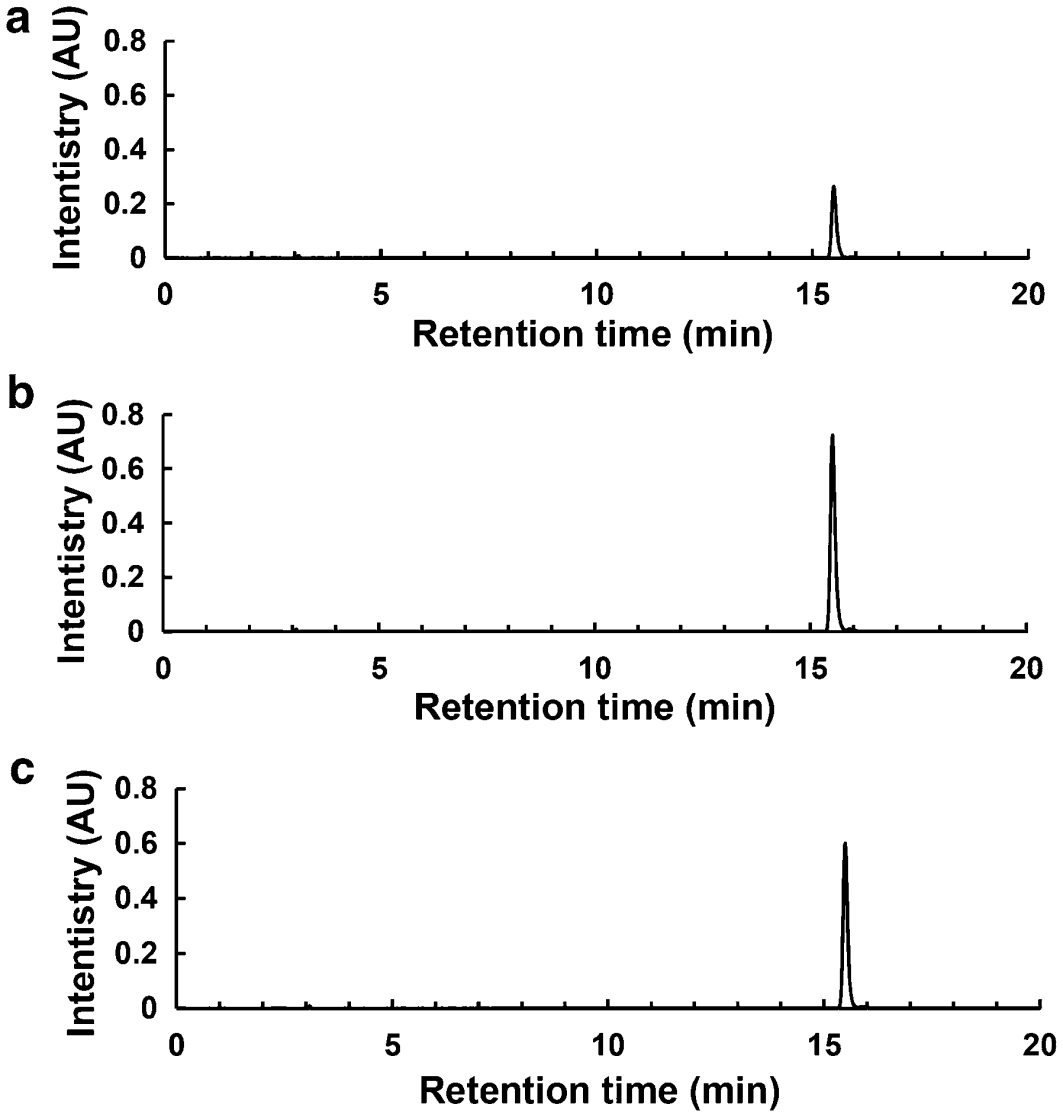
Enhanced CP production by co-culturing with bacteria B04 or B15. **a** The chromatogram of CP production of *Cercospora* sp. JNU001 alone. **b**, **c** The chromatogram of enhanced CP production by co-culturing *Cercospora* sp. JNU001 with B04 (**b**) or B15 (**c**)

As co-cultivation often enhances the production of metabolites by mimicking natural situations [29, 36], we began with screening the endophytic bacteria community related to LSD to mimic the phenomenon caused by pathogen *Cercospora* sp., and then co-cultured each of them with *Cercospora* sp. JNU001 to further increase CP production. After extensive purification, a total of 16 pure bacteria were isolated from the LSD related leaves (Additional file 1: Table S1). Next, each of them was co-cultured with *Cercospora* sp. JNU001 to investigate their effect on the production of CP under the above optimized conditions. It was found that most of them had a negative effect on its production from 92.72 to 10.84% (Additional file 1: Table S1). The B10 strain even caused the death of *Cercospora* sp. JNU001 (Additional file 1: Table S1). To our delight, B04 and B15 strains had a positive effect to increase the production of CP (Fig. 2 and Additional file 1: Table S1). Furthermore, ^1^H NMR analysis showed that the product purified from co-cultivation was still CP (Additional file 1: Fig. S1). Thus, these results allowed us to further characterize B04 and B15 strains and then investigated how they improved the production of CP.

### Identification and characterization of B04 and B15 strains

**Fig. 3:**
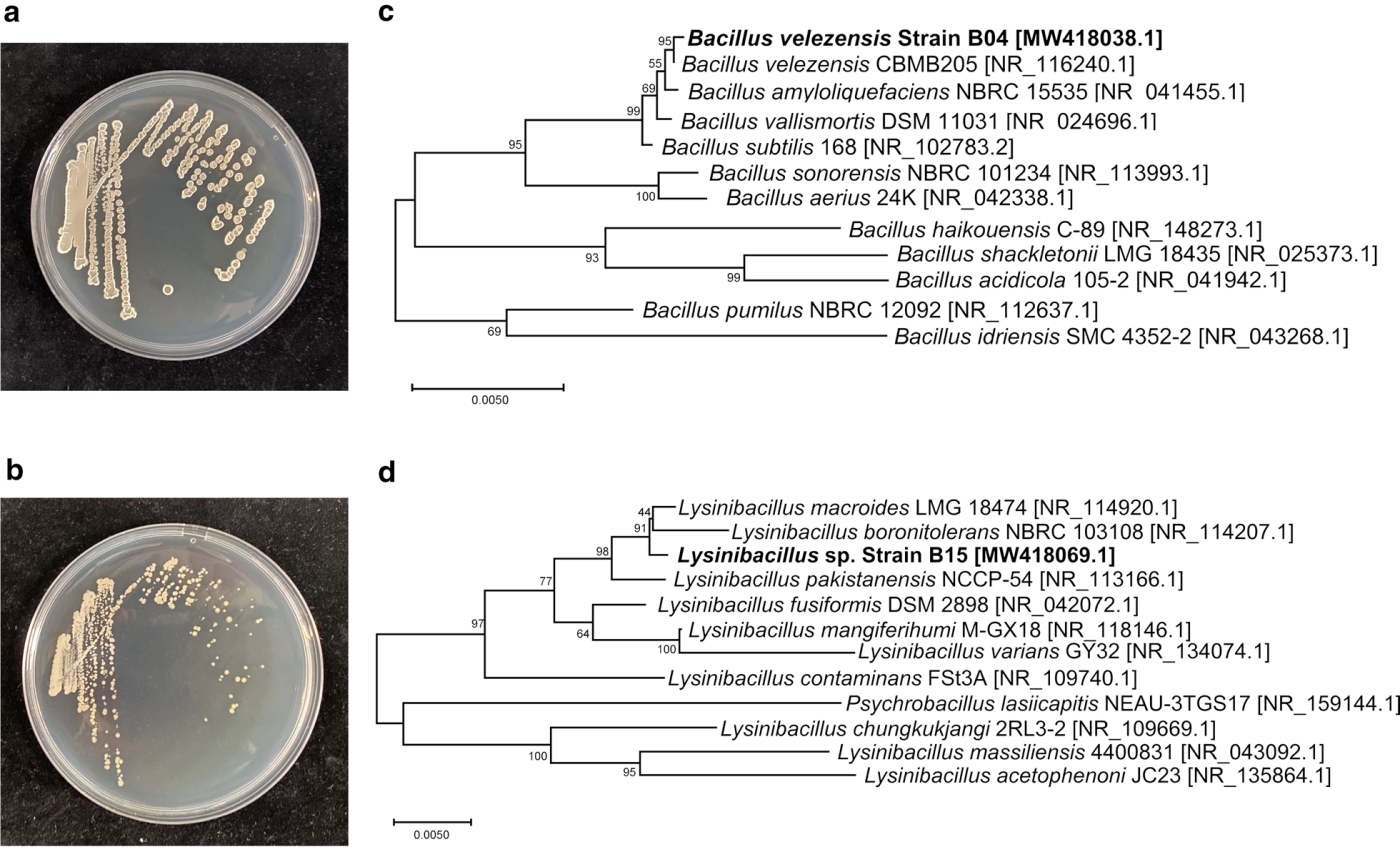
Identification and characterization of two isolated bacteria B04 and B15. **a**, **b** Microscopic appearance of culture colony of B04 strain and B15 strain on LB plate after 24 h. **c** The phylogenetic tree of B04 strain and its relationship with other *Bacillus* species. **d** The phylogenetic tree of B15 strain and its relationship with other *Lysinibacillus* species

It showed that the B04 colony appeared round, rough and white in color (Fig. 3a), while the B15 colony appeared round, smooth, small and white in color (Fig. 3b), suggesting that different molecular mechanisms might be applied by B04 and B15 to increase the production of CP. Based on the analysis of 16S rDNA nucleotide sequences (GenBank accession number MW418038.1 for B04, MW418069.1 for B15, respectively), the phylogenetic tree for B04 or B15 strain was established through the alignment and cladistics analysis of homologous nucleotide sequence (Fig. 3c, d). B04 strain and B15 strain belonged to *Bacillus velezensis* and *Lysinibacillus* sp., respectively. Compared to *Bacillus velezensis* CBMB205 (GenBank accession number, NR_116240.1), B04 strain has a similarity of 99.57%, which was then designated as *Bacillus velezensis* B04 (Fig. 3c). B15 strain shows a similarity of 99.43% with *Lysinibacillus macroides* LMG18474 (GenBank accession number, NR_114920.1) (Fig. 3d), and then was named as *Lysinibacillus* sp. B15.

### Optimization of co-culture conditions to enhance CP production

**Fig. 4:**
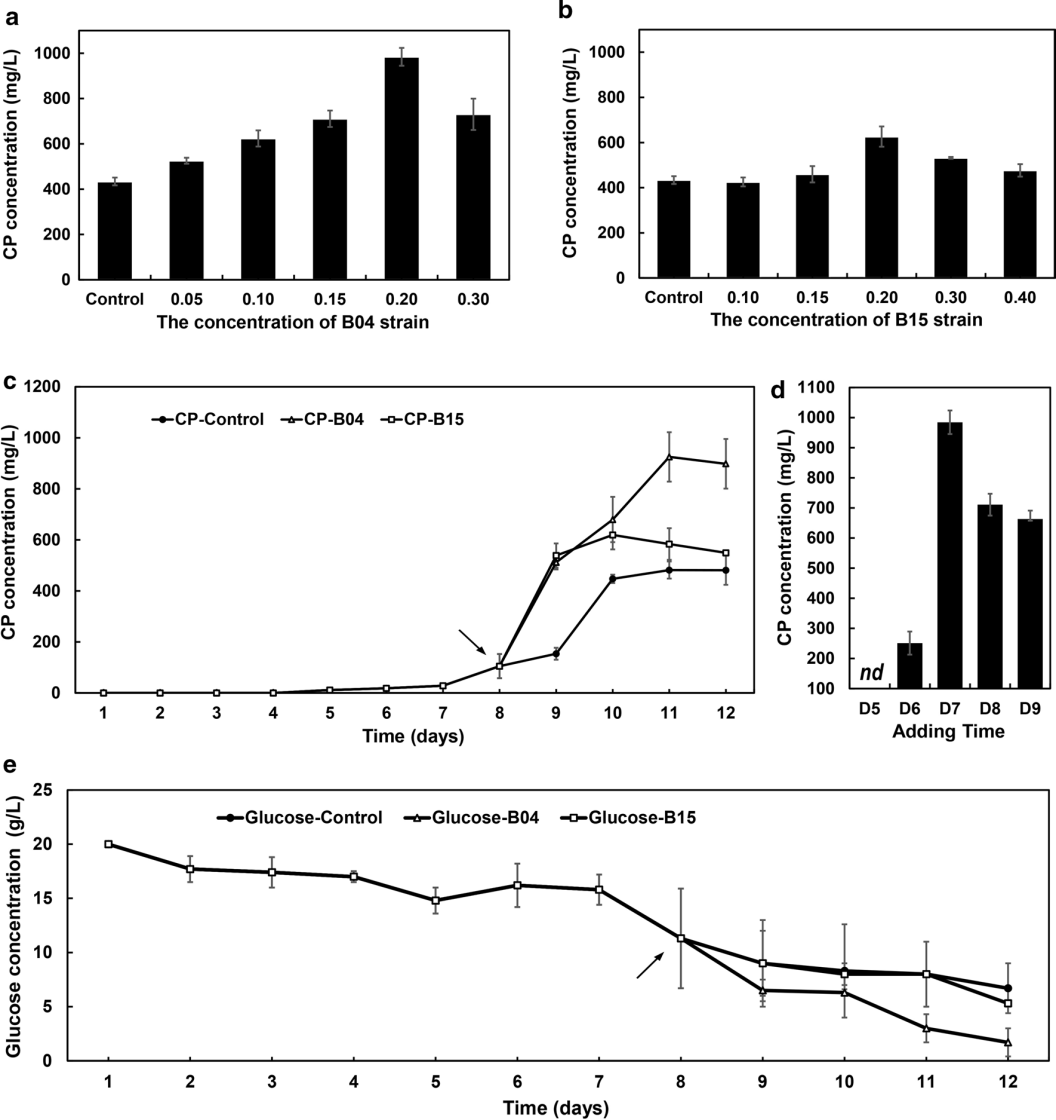
Effect of CP production by co-culturing B04 or B15 strain with *Cercospora* sp. JNU001. **a**, **b** Effect of the concentration of B04 or B15 strain on CP production. **c.** Effect of culture time on CP production when *Cercospora* sp. JNU001 was co-cultured with B04 or B15. **d** Effect of the adding time of B04 on CP production. **e** The glucose consumption of *Cercospora* sp. JNU001 grew in the modified S-7 medium and under co-culture with B04 or B15 strain condition. The arrows indicate the beginning of significant differences caused by co-culture

Next, we optimized co-culture conditions by adding different amounts of B04 or B15 to *Cercospora* sp. JNU001 pre-cultures, which initially grew overnight and then was diluted to different concentrations with the modified S-7 medium (Fig. 4a, b). It showed that B04 could greatly enhance the production of CP at different concentrations compared with the condition without co-culturing with B04 in modified S-7 medium, and the CP production reached a maximum of 984.4 mg/L when B04 was added at the final concentration of 0.20 OD_600_ (Fig. 4a), which was 2.67-fold and 7.68-fold higher than the one in modified S-7 medium and the original condition [35], respectively. However, its production decreased when more B04 was added (Fig. 4a). Interestingly, the production of CP only increased when the B15 strain was added around 0.20 OD_600_, in which the highest production of CP was achieved at 626.3 mg/L, which was 1.33-fold of that in modified S-7 medium (Fig. 4b). No significant increase was observed when less or more B15 strain was used (Fig. 4b).

To better understand the effect of co-culturing *Cercospora* sp. JNU001 with B04 or B15 strain, their time-course of CP production were analyzed. It showed that the amount of CP was very low at the beginning phase (Fig. 4c), and then started to secrete more CP after day 7. Under the control strain, the production of CP reached the maximum at day 11 (Fig. 4c), similar with the unmodified S-7 medium (Fig. 1b). Considering the growing speed of bacteria and *Cercospora* sp. JNU001 and the antimicrobial activity of CP, the appropriate time to add B04 or B15 was around day 7, in which *Cercospora* sp. JNU001 began to produce CP and then could use its antimicrobial activity to introduce the growth pressure and competition for bacteria. To verify the hypothesis, the effect of the adding time of B04 (day 5, 6, 7, 8 and 9) was analyzed. Surprisingly, no CP was detected when B04 was added at day 5 (Fig. 4d). The production of CP was impaired when B04 was added at day 6. Interestingly, although the amount of CP was enhanced when the B04 strain was added at day 8 and 9, the production of CP was significantly impaired when compared with the condition at day 7 (Fig. 4d), illustrating that the optimal time to add B04 was day 7. Moreover, the maximum of CP production also happened at day 11 (Fig. 4c). After day 11, *Cercospora* sp. JNU001 appeared to autolyze and had a negative effect on CP production (Additional file 1: Fig. S2). Similarly, the same phenomenon was observed for the strain B15 (Fig. 4c).

To further support the aforementioned conclusions, we also investigated the glucose utilization by measuring the remaining glucose concentration during the time-course of fermentation (Fig. 4e). It clearly showed that the glucose utilization was greatly increased after day 7 no matter with or without co-culturing with B04 or B15 (Fig. 4e), which was well correlated with the production of CP (Fig. 4c). Interestingly, the glucose utilization was similar under control condition and B15 co-culture condition, but more glucose was consumed under B04 co-culture condition after day 7, probably owing to the requirement of more energy to synthesize CP as it delivered much more CP than the other two conditions. Moreover, the remaining glucose was very limited after day 12 under B04 co-culture condition, which could explain the autolysis of *Cercospora* sp. JNU001 (Additional file 1: Fig. S2).

### Effect of live B04 and B15 on fungal growth and cercosporin secretion

**Fig. 5:**
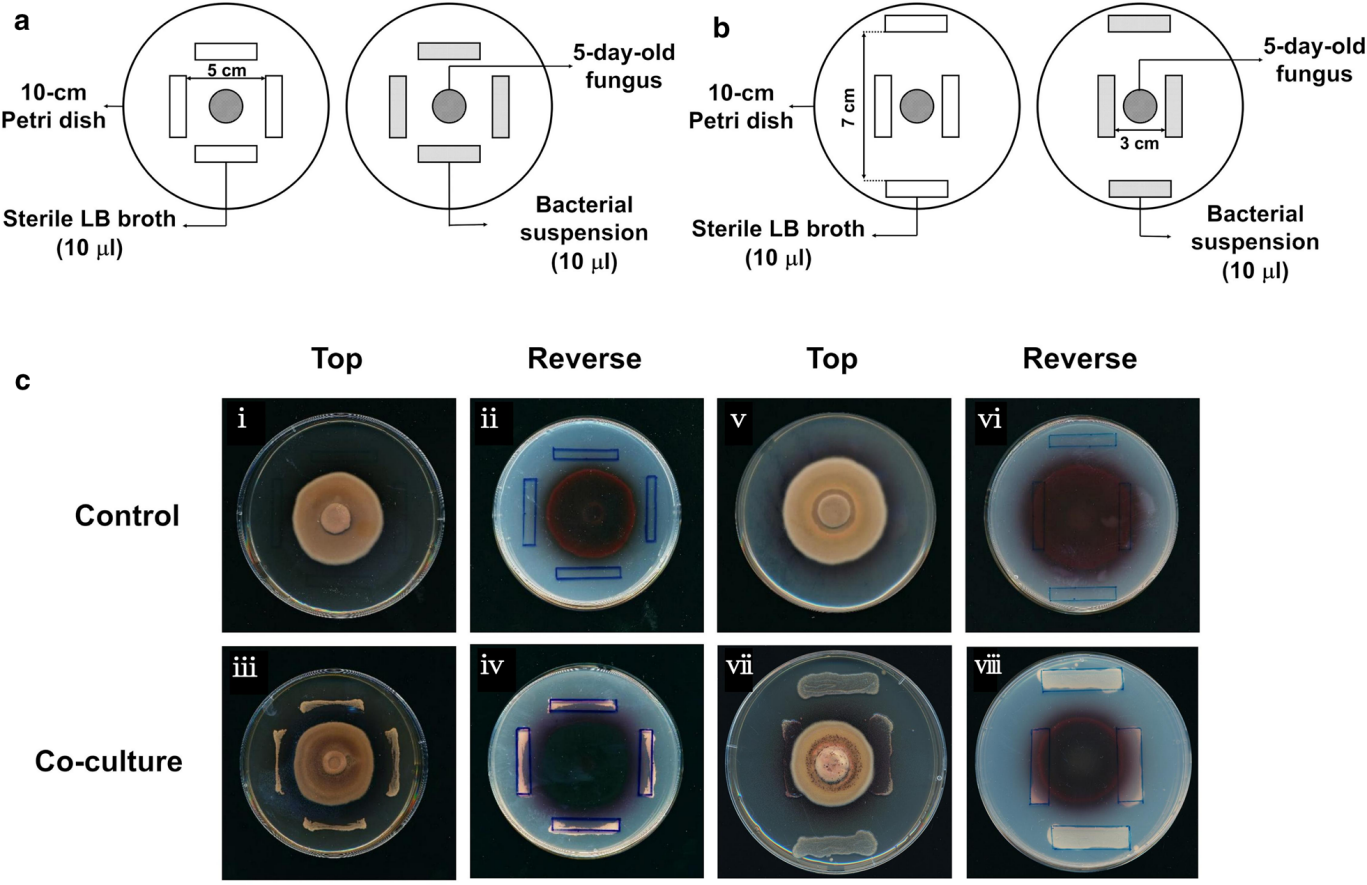
*In vitro* confrontation bioassays between bacteria and *Cercospora* sp. JNU001. **a** Schematic diagram of in vitro confrontation bioassay between B04 and *Cercospora* sp. JNU001. **b** Schematic diagram of in vitro confrontation bioassay between B15 and *Cercospora* sp. JNU001. **c** Effects of B04 (i–iv) or B15 (v–viii) on *Cercospora* sp. JNU001

To understand molecular mechanisms that improved the production of CP by B04 or B15 strain, we next performed in vitro fungal-bacterial confrontation bioassays (Fig. 5a, b) [37, 38]. It showed that B04 and B15 strains resulted in different phenomena (Fig. 5c). Surprisingly, *Cercospora* sp. JNU001 was unable to cross the boundary of the B04 strain (Fig. 5c(i–iv)), but it clearly induced the secretion of CP as it was well distributed outside of the boundary of *Cercospora* sp. JNU001 with the disappearance of the red ring of CP (Fig. 5c(iv)), in which the growth of both of B04 and *Cercospora* sp. JNU001 was somehow inhibited (Fig. 5c(iii, iv)). On the contrary, *Cercospora* sp. JNU001 obviously crossed over the boundary of B15 (Fig. 5c(v–viii)). Moreover, the red ring of CP still existed and B15 bacteria also became red once they got contacted with *Cercospora* sp. JNU001 (Fig. 5c(viii)), suggesting that B15 had the ability to adsorb CP to stimulate its secretion and then enhance its production. To verify this hypothesis, we then investigated whether B15 could emit the red fluorescence from CP after co-culturing with *Cercospora* sp. JNU001. As expected, the B15 strain alone did not show any fluorescence, but became red after co-culturing with *Cercospora* sp. JNU001 (Additional file 1: Fig. S3), confirming that CP could be adsorbed by B15 strain to facilitate its production, which was supported by other study that the adsorption ability could enhance the production of metabolites [39]. In fact, this result was also supported by our own investigation, in which the production of CP was improved with the yield of 787.9 mg/L when XAD-16 resins, the macroporous materials with excellent potential adsorption capacity, were used as a substitute to replace B15 strain (Additional file 1: Fig. S4).

**Fig. 6:**
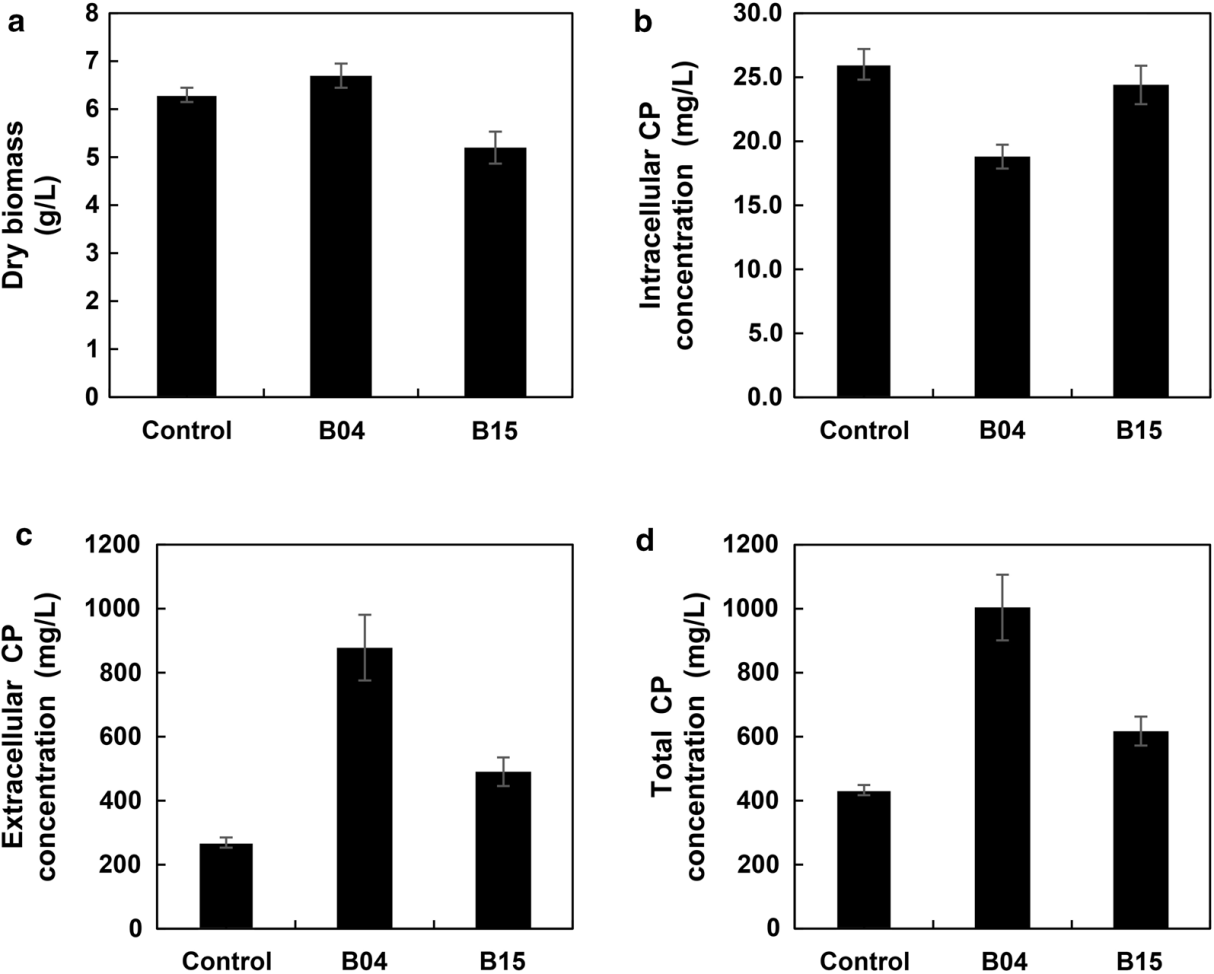
Effects of B04 and B15 on fungal growth and CP secretion. **a** Dry fungal biomass of *Cercospora* sp. JNU001 without or with bacteria. **b** Intracellular CP production of *Cercospora* sp. JNU001 extracted from **a**. **c** Extracellular CP production purified from culture broth after co-culture without or with bacteria. **d** Total CP production, which was calculated by intracellular CP production (**b**) and extracellular CP production (**c**)

Next, we investigated whether the above phenomena would also happen in the liquid fermentation condition. It was found that there was no obvious difference of dry biomass between the control strain and B04 co-culture condition (Fig. 6a), suggesting that there was no influence on fungal growth under B04 co-culture conditions. However, the dry biomass of *Cercospora* sp. JNU001 was slightly decreased when it was co-cultured with B15. Interestingly, the amount of CP extracted from the dry biomass of B15 co-culture was similar with the control strain, but slightly decreased in the B04 co-culture system (Fig. 6b), probably due to the excellent secretion ability of CP induced by B04 (Fig. 5c(iv), 6c). As the proportion of CP secreted into the culture broth was higher than the one extracted from dry biomass in both B04 and B15 co-culture conditions, which mainly contributed to the production of CP (Fig. 6d), it was suggested that both of B04 and B15 could facilitate CP secretion to improve its production. Furthermore, the secretion ability of CP induced by the B04 strain was much better than the B15 strain (Fig. 6c), implying that B04 and B15 might employ two different mechanisms to improve the production of CP.

### Morphological observation of co-culture samples

**Fig. 7:**
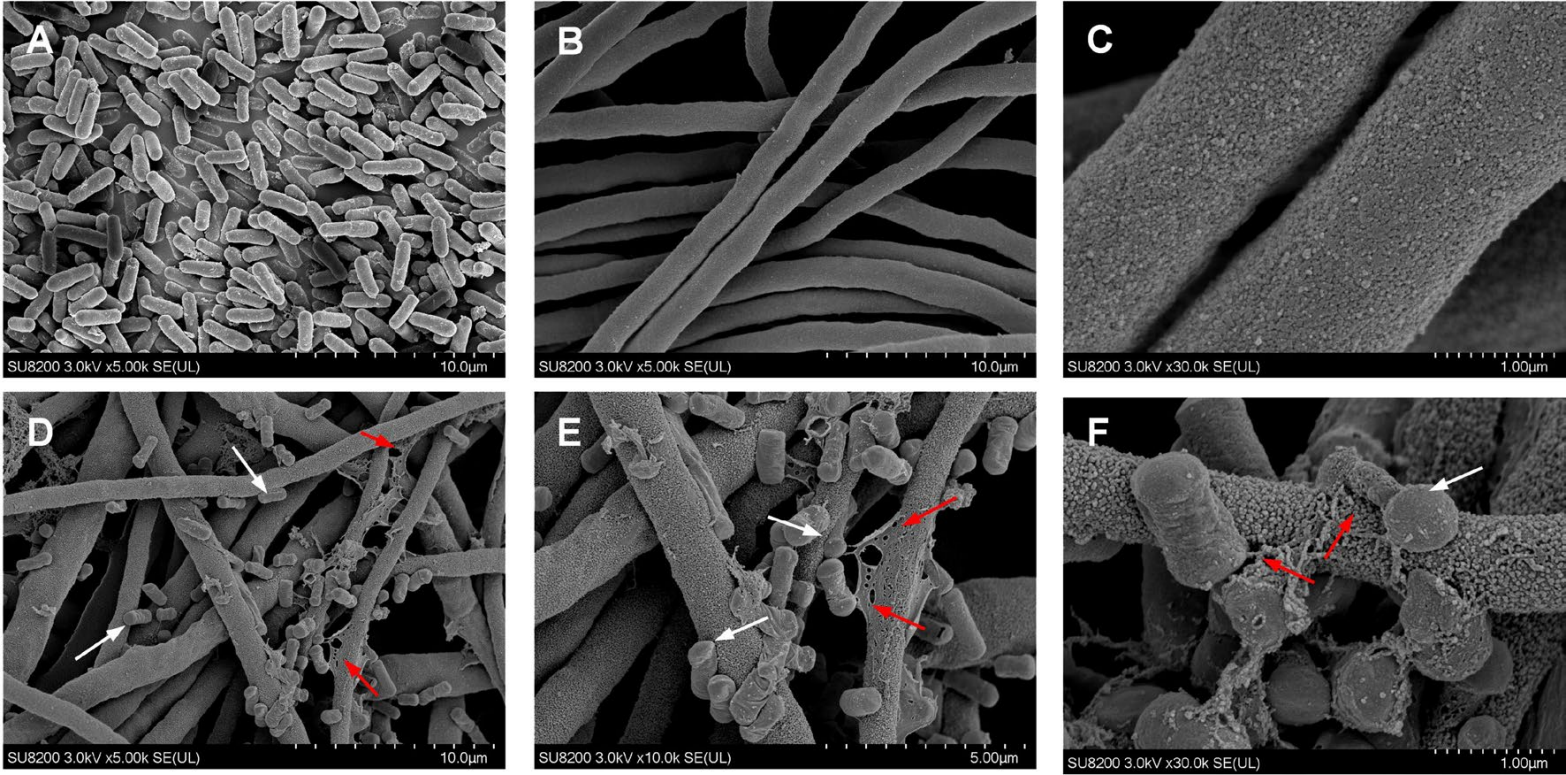
FESEM observation of co-culture *Cercospora* sp. JNU001 with B04. **a** B04 samples. **b**, **c** *Cercospora* sp. JNU001 samples. **d–f** Co-culture samples of B04 and *Cercospora* sp. JNU001. White arrows indicate bacteria B04, and red arrows indicate the damage of fungal hyphae. Scale bar was indicated

To further support the above conclusion that two different mechanisms were applied by B04 and B15 to increase the production of CP through enhancing its secretion ability, field emission scanning electron microscope (FESEM) was employed to investigate the morphology of co-culture samples (Fig. 7), which was derived from the optimized liquid fermentation. It showed that the bacteria B04 were attached on the fungal hyphae surface and seemed to have the capacity to loosen it (Fig. 7d–f), even to damage the fungal hyphae (Fig. 7e, f), which was very tight in the original *Cercospora* sp. JNU001 strain (Fig. 7b, c). To further support these phenomena, the Congo red differential medium with glucan, which is the main component of fungal cytoderm and can be degraded by glucanase [40–42], was employed to determine whether it would be degraded by B04. Indeed, the glucan around the B04 strain, but not B15 strain, was able to be degraded (Additional file 1: Fig. S5), indicating that B04 probably have an ability to secrete glucanase to loosen and damage the fungal hyphae [43], which could facilitate cercosporin secretion and then resulted in the improvement of CP production. To further support this notion, the commercial glucanase was directly added to *Cercospora* sp. JNU001 culture at day 7, and the CP production was indeed increased with the yield of 719.7 mg/L (Additional file 1: Fig. S6). Furthermore, it showed that bacteria B04 were somehow shrunk and became unhealthy when compared with the untreated ones (Fig. 7a, d–f).

However, as for the B15 co-culture condition, no obvious appearance change of *Cercospora* sp. JNU001 was observed, and only a few bacteria B15 had physical attachment on the surface of hyphae (Additional file 1: Fig. S7), in which the shape of B15 bacteria was also deformed like the bacteria B04.

Together with the result that CP was adsorbed and inserted into B15 bacteria (Additional file 1: Fig. S3), we concluded that bacteria B04 and B15 employed different mechanisms to enhance the production of CP, in which B04 could loosen and damage the hyphae of *Cercospora* sp. JNU001 to facilitate CP secretion while B15 had an ability to adsorb CP to improve its secretion. Surprisingly, the production of CP was not further improved but inhibited with the yield of 280.4 ± 2.9 mg/L when both of B04 and B15 were co-cultured with *Cercospora* sp. JNU001.

## Conclusions

To improve the production of CP, besides traditional optimization methods, including optimization of culture medium and culture conditions, the co-culture method was also employed in this study. After extensive screening and purification, two new identified bacteria *Bacillus velezensis* B04 and *Lysinibacillus* sp. B15 further enhanced the production of CP by separately co-culturing with fungus *Cercospora* sp. JNU001 with the yield of 984.4 mg/L and 626.3 mg/L, which were 7.68-fold and 4.89-fold of that in the starting condition, respectively. Moreover, two different mechanisms were found to increase the production of CP by these two bacteria. *Bacillus velezensis* B04 had the ability to loosen or damage the surface of hyphae and then to improve the secretion ability of CP, while *Lysinibacillus* sp. B15 could adsorb and accumulate CP to increase CP production. Thus, we here provided a novel effective co-culture method to enhance CP production of *Cercospora* sp., which allows to develop more applications of CP.

## Materials and methods

### CP-producing fungal strain and culture conditions

CP was produced by an endophytic fungus *Cercospora* sp. JNU001, which was isolated from the tree bark of *Taxus chinensis* in Lishui, Zhejiang, China and reserved in China Center for Type Culture Collection (CCTCC 2017842). The strain was stored on modified S-7 solid medium (glucose: 20 g/L, sodium acetate: 1 g/L, soy peptone: 2 g/L, phenylalanine: 5 mg/L; sodium benzoate: 100 mg/L, 1 M KH_2_PO_4_ buffer: 1 mL, biotin: 1 mg/L, Ca(NO_3_)_2_: 6.5 mg/L, pyridoxal: 1 mg/L, calcium pantothenate: 1 mg/L, thiamine: 1 mg/L, MnCl_2_: 5 mg/L, FeCl_3_: 2 mg/L, Cu(NO_3_)_2_: 1 mg/L, MgSO_4_: 3.6 mg/L, ZnSO_4_: 2.5 mg/L, agar powder: 15 g/L) at 4 °C, or in cryotubes with glycerol (20%) at − 80 °C.

The traditional S-7 medium before optimization was shown below: Glucose: 1 g/L, fructose: 2 g/L, saccharose: 6 g/L sodium acetate: 1 g/L, soy peptone: 2 g/L, phenylalanine: 5 mg/L; sodium benzoate: 100 mg/L, 1 M KH_2_PO_4_ buffer: 1 mL, biotin: 1 mg/L, Ca(NO_3_)_2_: 6.5 mg/L, pyridoxal: 1 mg/L, calcium pantothenate: 1 mg/L, thiamine: 1 mg/L, MnCl_2_: 5 mg/L, FeCl_3_: 2 mg/L, Cu(NO_3_)_2_: 1 mg/L, MgSO_4_: 3.6 mg/L, ZnSO_4_: 2.5 mg/L, agar powder: 15 g/L.

*Cercospora* sp. JNU001 was inoculated into 500 mL flasks with 100 mL modified S-7 liquid medium and then cultured at 25 °C in a shaker (ZQZY-AF8, Zhichu, China) with 135 rpm upon continuous light illumination (flasks were fixed 16.67 ± 3.00 cm away from a 15 W compact fluorescent lamp (CFL)).

### Separation and purification of CP

50 mL dichloromethane (DCM) was added to the fermentation broth after 11 days and then flasks were put back to the shaker at 135 rpm for 36 h to ensure the complete extraction of CP. This procedure was repeated twice. The organic phase containing CP was collected and DCM was evaporated (RV8, IKA, Germany) to obtain the raw CP, which was then dissolved in methanol and purified by a Sephadex column LH-20.

### Quantitative determination of CP production and glucose consumption

To rapidly determine the content of CP, the raw CP was analyzed by HPLC (2695, Waters, America) using an Aglient column (5 μm, C_18_, 100 Å, 4.6 × 250 mm) with the characteristic adsorption wavelength of CP at 472 nm. The purified CP was used as a reference. Then, the concentration of CP of each sample could be calculated. To study the time-course of CP production, 2 mL of culture broth was taken out at each day and extracted three times by 2 mL DCM. The extracted fraction was collected and DCM was evaporated. The raw materials with CP was dissolved in 200 µL methanol and loaded into HPLC. The amount of CP was detected by HPLC. The gradient was as follows: 0–20 min, 20–85% Solvent A; 20–23 min, 85% Solvent A; 23–27 min, 85–20% Solvent A; 27–30 min, 20% Solvent A. Solvent A: HPLC grade acetonitrile containing 0.5% formic acid; Solvent B: HPLC grade H_2_O containing 0.5% formic acid. The solvent flow rate was 1.0 mL/min. The column was pre-equilibrated for 10 min between injections.

To study the glucose consumption, 100 µL culture broth was taken out from the flask each day and diluted with double distilled water (dd H_2_O). The diluted broth was centrifuged at 12,000 rpm to remove bacterium and fungal mycelia, and then injected to SBA-40E Biosensor Analyzer, which was made by Institute of Biology, Shandong Academy of Science, to measure the glucose concentration.

### Isolation and identification of leaf-spot-disease-related endophytic bacteria

Leaves with LSD was collected from August to October in Wuxi, Jiangsu, China. All leaf samples were immediately stored in sealed bags on ice and then sterilized as the following procedures [44]. The fresh leaves were washed by tap water for 1–2 h and cut into small pieces (2–3 cm long and 0.5 cm wide), which were then rinsed by sterilized water for 3 times, dipped in 75% ethyl alcohol for 1 min and again rinsed by sterilized water for 3 times. Next, some new wounds of leaf pieces were made by the sterilized scalpel to contact LB agar plate (tryptone: 10 g/L, yeast extra: 5 g/L, sodium chloride: 10 g/L, agar powder: 15 g/L, pH: 7.0–7.2) after drying with sterilized filter tissue paper and then cultured at 37 °C without light for 48 h. The single colony was obtained by streak plate method, and then inoculated into LB medium to cultivate without light at 37 °C in a shaker at 200 rpm for 24 h. Each of purified bacteria was stored in cryovial tubes with glycerol (25%) at − 80 °C.

To characterize each of purified bacteria, the bacterial general primers, 27F (5′-AGAGTTTGATCATGGCTCAG-3′) and 1492R (5′-TACGGCTACCTTG TTACGACTT-3′) were used to amplify the 16S rDNA of each of them. The PCR reaction was performed in a final volume of 50 µL: 1 µL bacteria culture medium containing DNA, 25 µL of 2 × Premix, 1 µL of 27F (5 nmol/L) and 1 µL of 1492R (5 nmol/L), 22 µL double distilled water (ddH_2_O). The amplified PCR product was purified and sequenced by GENEWIZ Inc. (Suzhou, China). The obtained sequences were uploaded to GenBank in NCBI database and the cladogram was constructed by neighbor-joining method (1000-replicate bootstrap) in MEGA-X (version 10.1.8) after homologous comparisons with the existed bacterial sequences in NCBI. The scale bar of the cladogram indicated the number of base substitutions per site.

### Establishment of co-culture conditions

To improve the production of CP, two small pieces (5 mm × 5 mm) of *Cercospora* sp. JNU001 from the modified S-7 agar plate were firstly inoculated into 100 mL modified S-7 liquid medium at 25 °C on a rotating shaker at 135 rpm for 7 days. At day 6, the single colony of each of two isolated bacteria B04 and B15 were inoculated into 50 mL LB medium and cultured at 37 °C on a rotating shaker at 200 rpm overnight. At day 7, the customized number of bacteria cells was harvested, centrifuged and resuspended by modified S-7 liquid medium, and then added into pre-culture *Cercospora* sp. JNU001. The co-culture samples grew for another 3-5 days at 25 °C on a rotating shaker at 135 rpm with continuous light illumination (flasks were fixed 16.67 ± 3.00 cm away from a 15 W compact fluorescent lamp (CFL)). After that, 50 mL DCM was added into culture broth to extract CP using the aforementioned method. The dry biomass was measured immediately after treatment in vacuum freeze dryer (FreeZone 6 Plus, LABCONCO, America) for 3 days. Similarly, 50 mL DCM was also used to extract CP from dry biomass of *Cercospora* sp. JNU001. The content of CP was detected by HPLC.

### In vitro fungal-bacterial confrontation bioassay

Based on the method reported by Wang et al. [38], different kinds of confrontation bioassays (in vitro) were conducted between *Cercospora* sp. JNU001 and each of two isolated bacteria. Firstly, a small piece (5 mm × 5 mm) of marginal mycelium of *Cercospora* sp. JNU001 with agar was dug out and reset in the center of a 10 cm modified S-7 agar plate and let it grow for 5 days. Next, the single colony of different bacteria was inoculated in LB medium at 37 °C on a rotating shaker at 200 rpm for 24 h on day 5, respectively. As it is shown in Fig. 5, bacterial suspension (10 µL) was streaked in four parallel rectangular areas (approximately 3 cm × 0.5 cm) and cultured for another 10 days. The morphology was observed.

### Morphological observation

The physical attachment between *Cercospora* sp. JNU001 and B04 or B15 was observed by FESEM (SU-8220, Hitachi, Japan). The samples were collected at day 11 (3 days after co-culturing). Meanwhile, pure bacteria and fungus were also observed as a control. To study whether B15 had an ability to adsorb CP, B15 samples were collected from in vitro fungal-bacterial confrontation assays when *Cercospora* sp. JNU001 partially reached the edge of the rectangular area of B15, and then dissolved by double distilled water. 10 µL was dropping onto a glass slide and measured by a fluorescence microscope (CKX53, OLYMPUS, Japan) as CP has the capacity to emit the fluorescence. Meanwhile, B15 samples far from *Cercospora* sp. JNU001 on the in vitro fungal-bacterial confrontation assays were also collected and used as a control.

### *In*-*situ* adsorption of XAD-16 resins on CP

To investigate whether XAD-16 resins had the similar effect like B15 strain on enhanced production of CP, XAD-16 resins (30 g/L), which was pretreated as other reports [45], were added to *Cercospora* sp. JNU001 cultures at day 7. CP was then extracted by DCM, resolved in methanol and analyzed by HPLC.

### Congo red stain

To study whether B04 or B15 had an ability to secret glucanase to damage the fungal hyphae, Congo red agar plate (0.05% K_2_HPO_4_, 0.05% MgSO_4_, 0.05% NaCl, 0.2% (NH_4_)_2_SO_4_, 0.5% glucan, 0.2% Congo red and 1.5% agar) was prepared. Next, B04 or B15 on LB plate was dug out, transferred onto the center of glucan Congo red agar plate, and let it grow for another 4-5 days. The transparent zone was scanned.

## Supplementary Information

**Additional file 1: Table S1.** 16 bacteria isolated from leaves with leaf spot diseases and their effects on CP production by co-cultivation. **Figure S1.** ^1^H NMR of CP structure. **Figure S2.** Autolysis of *Cercospora* sp. JNU001. **Figure S3.** Fluorescence microscope observation of B15 after culturing with *Cercospora* sp. JNU001. **Figure S4.** Effect of XAD-16 resins on its CP production. **Figure S5.** Analysis of the ability of B04 and B15 to degrade glucan. **Figure S6.** Influence of glucanase on CP production. **Figure S7.** FESEM observation of *Cercospora* sp. JNU001 with B15.

## Data Availability

All data generated or analyzed during this study are included in this published article and its Additional file.

## References

[1] Daub ME (1982). Cercosporin, a photosensitizing toxin from *Cercospora* species. Phytopathol..

[2] Fajola AO (1978). Cercosporin, a phytotoxin from *Cercospora* spp. Physiol Plant Pathol..

[3] Kuyama S, Tamura T (1957). Cercosporin. A pigment of *Cercosporina Kikuchii Matsumoto et Tomoyasu.* I. Cultivation of fungus, isolation and purification of pigment. J Am Chem Soc.

[4] Chupp TC (1948). Notes on some *Cercosporae* of India. Mycologia.

[5] Reznikov S, De Lisi V, Claps P, González V, Devani MR, Castagnaro AP, Ploper LD (2019). Evaluation of the efficacy and application timing of different fungicides for management of soybean foliar diseases in northwestern Argentina. Crop Prot..

[6] Crous PW, Groenewald JZ, Groenewald M, Caldwell P, Braun U (2006). Species of *Cercospora* associated with grey leaf spot of maize. Stud Mycol.

[7] Avila A, Groenewald JZ, Trapero A, Crous PW (2005). Characterisation and epitypification of *Pseudocercospora cladosporioides*, the causal organism of *Cercospora* leaf spot of olives. Mycol Res.

[8] Mulrooney CA, O’Brien EM, Morgan BJ, Kozlowski MC (2012). Perylenequinones: isolation, synthesis, and biological activity. Eur J Org Chem.

[9] Daub ME (1987). Resistance of fungi to the photosensitizing toxin, cercosporin. Phytopathol..

[10] Daub ME, Briggs SP (1983). Changes in tobacco cell membrane composition and structure caused by cercosporin. Plant Physiol.

[11] Daub ME, Ehrenshaft M (2000). The photoactivated *Cercospora* toxin cercosporin: contributions to plant disease and fundamental Biology. Annu Rev Phytopathol.

[12] Diwu Z, Lown JW (1993). Photosensitization with anticancer agents: 15. Perylenequinonoid pigments as potential photodynamic therapeutic agents: formation of semiquinone radicals and reactive oxygen species on illumination. J Photochem Photobiol B..

[13] Guedes RC, Eriksson LA (2007). Photophysics, photochemistry, and reactivity: molecular aspects of perylenequinone reactions. Photochem Photobiol Sci.

[14] Dobrowolski D, Foote C (2010). Cercosporin, a singlet oxygen generator. Angew Chem Int Ed.

[15] Kumarihamy M, Khan SI, Jacob M, Tekwani BL, Duke SO, Ferreira D, Nanayakkara NP (2012). Antiprotozoal and antimicrobial compounds from the plant pathogen *Septoria pistaciarum*. J Nat Prod.

[16] Mastrangelopoulou M, Grigalavicius M, Berg K, Ménard M, Theodossiou TA (2019). Cytotoxic and photocytotoxic effects of cercosporin on human tumor cell lines. Photochem Photobiol Sci.

[17] Grigalavicius M, Mastrangelopoulou M, Arous D, Juzeniene A, Ménard M, Skarpen E, Berg K, Theodossiou TA (2020). Photodynamic efficacy of cercosporin in 3D tumor cell cultures. Photochem Photobiol.

[18] Morgan BJ, Dey S, Johnson SW, Kozlowski MC (2009). Design, synthesis, and investigation of protein kinase C inhibitors: total syntheses of (+)-calphostin D, (+)-phleichrome, cercosporin, and new photoactive perylenequinones. J Am Chem Soc.

[19] Li J, Bao W, Tang Z, Guo B, Zhang S, Liu H, Huang S, Zhang Y, Rao Y (2019). Cercosporin-bioinspired selective photooxidation reactions under mild conditions. Green Chem.

[20] Li J, Bao W, Zhang Y, Rao Y (2019). Metal-free fercosporin-photocatalyzed C-S coupling for the selective synthesis of aryl sulfides under mild conditions. Eur J Org Chem..

[21] Tang Z, Li J, Lin F, Bao W, Zhang S, Guo B, Huang S, Zhang Y, Rao Y (2019). Cercosporin-bioinspired photoreductive activation of aryl halides under mild conditions. J Catal.

[22] Zhang S, Tang Z, Bao W, Li J, Guo B, Huang S, Zhang Y, Rao Y (2019). Perylenequinonoid-catalyzed photoredox activation for the direct arylation of (het)arenes with sunlight. Org Biomol Chem.

[23] Zhang Y, Cao Y, Lu L, Zhang S, Rao Y (2019). Perylenequinonoid-catalyzed [4 + 1]- and [4 + 2]-annulations of azoalkenes: photocatalytic access to 1,2,3-thiadiazole/1,4,5,6-tetrahydropyridazine derivatives. J Org Chem.

[24] Lu L, Zhang Y, Yuan Z, Xu J, Li M, Wu Y, Wang L, Huang S, Rao Y (2021). Easily fabricated HARCP/HAp photocatalyst for efficient and fast removal of tetracycline under natural sunlight. Chem Eng J.

[25] Djebali N, Gaamour N, Badri M, Aouani ME (2010). Optimizing growth and conidia production of *Cercospora medicaginis*. Phytopathol Mediterr..

[26] Jiménez MM, Bahena SM, Espinoza C (2010). Trigos n: isolation, characterization, and production of red pigment from *Cercospora piaropi* a biocontrol agent for Waterhyacinth. Mycopathologia.

[27] Lynch FJ, Geoghegan MJ (1977). Production of cercosporin by *Cercospora* species. Trans Br Mycol Soc..

[28] Dong J, Zhang X, Baol J, Xu X, Qi S (2014). Secondary metabolites of the co-culture of *Aspergillus* sp. SCSGAF 0076 and *Bacillus* sp. MNMCCE 001. Acta Microbiol Sin..

[29] Jennifer W, Hassan HM, Marcel J, Rainer E, Rateb ME (2017). Dual induction of new microbial secondary metabolites by fungal bacterial co-cultivation. Front Microbiol..

[30] Ma YJ, Zheng LP, Wang JW (2023). Bacteria associated with *Shiraia* fruiting bodies influence fungal production of hypocrellin A. Front Microbiol..

[31] Xu D, Wang L, Du C (2015). Progress in microbial co-culture–A review. Acta Microbiol Sin..

[32] Zhou L, Miao Z, Xu D, Yang Z, Du C (2017). Comparative analysis of co-culture and pure culture of antibacterial metabolites of *Brevibacillus laterosporus* BL-21 and *Bacillus subtilis* HNDF2. Chin Agric Sci Bull.

[33] Zhu Y, Liu J, Liu J, Du G, Zhou J, Chen J (2012). A high throughput method to screen companion bacterium for 2-keto-l-gulonic acid biosynthesis by co-culturing *Ketogulonicigenium vulgare*. Process Biochem.

[34] Stierle A, Strobel G, Stierle D (1993). Taxol and taxane production by *Taxomyces andreanae*, an endophytic fungus of *Pacific yew*. Science.

[35] Tang Z, Bao W, Guo B, Rao Y (2019). Screening, identification and fermentation optimization of a cercosporin-producing strain. Sciencepap Online..

[36] Marmann A, Aly A, Lin W, Wang B, Proksch P (2014). Co-cultivation—A powerful emerging tool for enhancing the chemical diversity of microorganisms. Mar Drugs..

[37] Ma YJ, Zheng LP, Wang JW (2019). Inducing perylenequinone production from a bambusicolous fungus *Shiraia* sp. S9 through co-culture with a fruiting body-associated bacterium *Pseudomonas fulva* SB1. Microb Cell Fact..

[38] Wang XM, Yang HW, Ren CG, Zheng HL, Dai CC (2015). Consequences of antagonistic interactions between endophytic fungus and bacterium on plant growth and defense responses in *Atractylodes lancea*. J BASIC MICROB..

[39] Mei J, Min H, Lü Z (2009). Enhanced biotransformation of l-phenylalanine to 2-phenylethanol using an in situ product adsorption technique. Process Biochem.

[40] Kasana RC, Salwan R, Dhar H, Dutt S, Gulati A (2008). A rapid and easy method for the detection of microbial cellulases on agar plates using Gram’s iodine. Curr Microbiol.

[41] Semedo MC, Karmali A, Fonseca L (2015). A high throughput colorimetric assay of β-1,3-d-glucans by Congo red dye. J Microbiol Methods.

[42] Wood PJ, Erfle JD, Teather RM (1988). Use of complex formation between Congo Red and polysaccharides in detection and assay of polysaccharide hydrolases. Methods Enzymol.

[43] Xu T, Zhu T, Li S (2016). β-1,3-1,4-glucanase gene from *Bacillus velezensis* ZJ20 exerts antifungal effect on plant pathogenic fungi. World J Microbiol Biotechnol.

[44] Song PY, Lan QY, Lu ZY (2012). Identification and phylogenetic analysis on endophytic bacteria isolated from *Taxus chinensis var mairei*. Biotechnol..

[45] Zheng Y, Wang D, Xu P, Shen W, Zhang Y (2019). In-situ adsorption kinetics of macroporous resin on natamycin. Food Mach..

