# Supplementary material for: Enhanced cercosporin production by co-culturing Cercospora sp. JNU001 with leaf-spot-disease-related endophytic bacteria
Source: Microb Cell Fact. 2021 May 15;20:100. doi: 10.1186/s12934-021-01587-2 (PMC8126151; doi:10.1186/s12934-021-01587-2)
Supplement: Supplementary file 1 — Additional file 1: Table S1. 16 bacteria isolated from leaves with leaf spot diseases and their effects on CP production by co-cultivation. Figure S1. 1H NMR of CP structure. Figure S2. Autolysis of Cercospora sp. JNU001. Figure S3. Fluorescence microscope observation of B15 after culturing with Cercospora sp. JNU001. Figure S4. Effect of XAD-16 resins on its CP production. Figure S5. Analysis of the ability of B04 and B15 to degrade glucan. Figure S6. Influence of glucanase on CP production. Figure S7. FESEM observation of Cercospora sp. JNU001 with B15. [file 12934_2021_1587_MOESM1_ESM.doc]

| Bacteria used in co-cultivation | CP production compared with the untreated *Cercospora* sp. JNU001 |
| --- | --- |
| B01 | 75.68% |
| B02 | 90.90% |
| B03 | 87.86% |
| **B04** | **134.15%** |
| B05 | 78.87% |
| B06 | 107.43% |
| B07 | 10.84% |
| B08 | 63.21% |
| B09 | 21.41% |
| B10^*^ | 0.00% |
| B11 | 103.11% |
| B12 | 44.61% |
| B13 | 92.72% |
| B14 | 35.65% |
| **B15** | **120.44%** |
| B16 | 101.14% |
| * B10 caused the death of *Cercospora* sp. JNU001. | |

**Table S1** 16 bacteria isolated from leaves with leaf spot diseases and their effects on CP production by co-cultivation


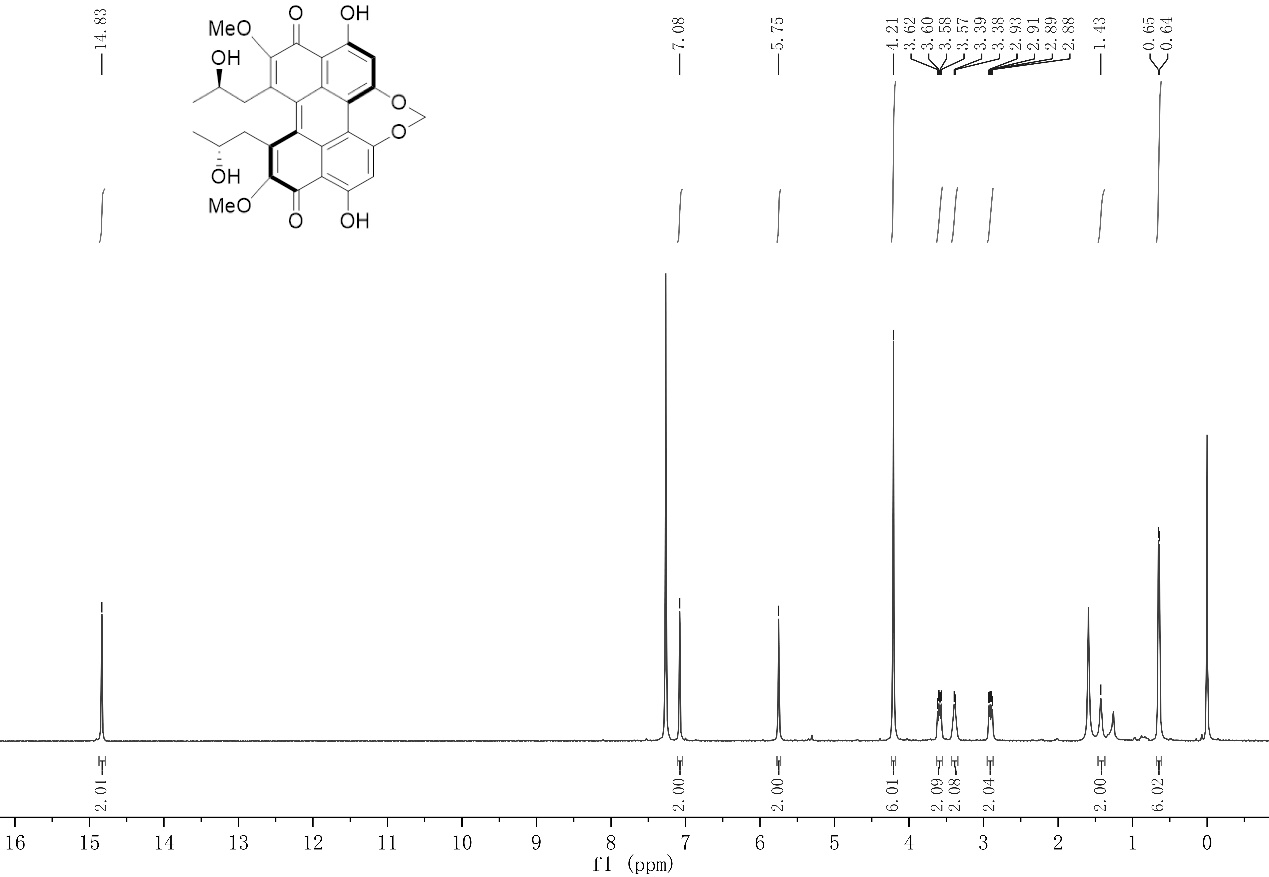


**Fig. S1** ^1^H NMR of CP structure.

Cercosporin identification: ^1^H NMR (400 MHz, CDCl_3_) δ ppm 14.82 (s, 2H, ArH), 7.06 (s, 2H, ArH), 5.57 (s, 2H, CH_2_), 4.20 (s, 6H, 2OCH_3_), 3.62-3.57 (m, 2H, CH_2_), 3.42-3.37 (m, 2H, CH_2_), 2.93-2.88 (m, 2H, CH_2_), 0.63 (d, 6H, *J* = 8 Hz, 2CH_3_). CP obtained from co-culture systems and *Cercospora* sp. JNU001 alone has the same structure as demonstrated by ^1^H NMR analysis.


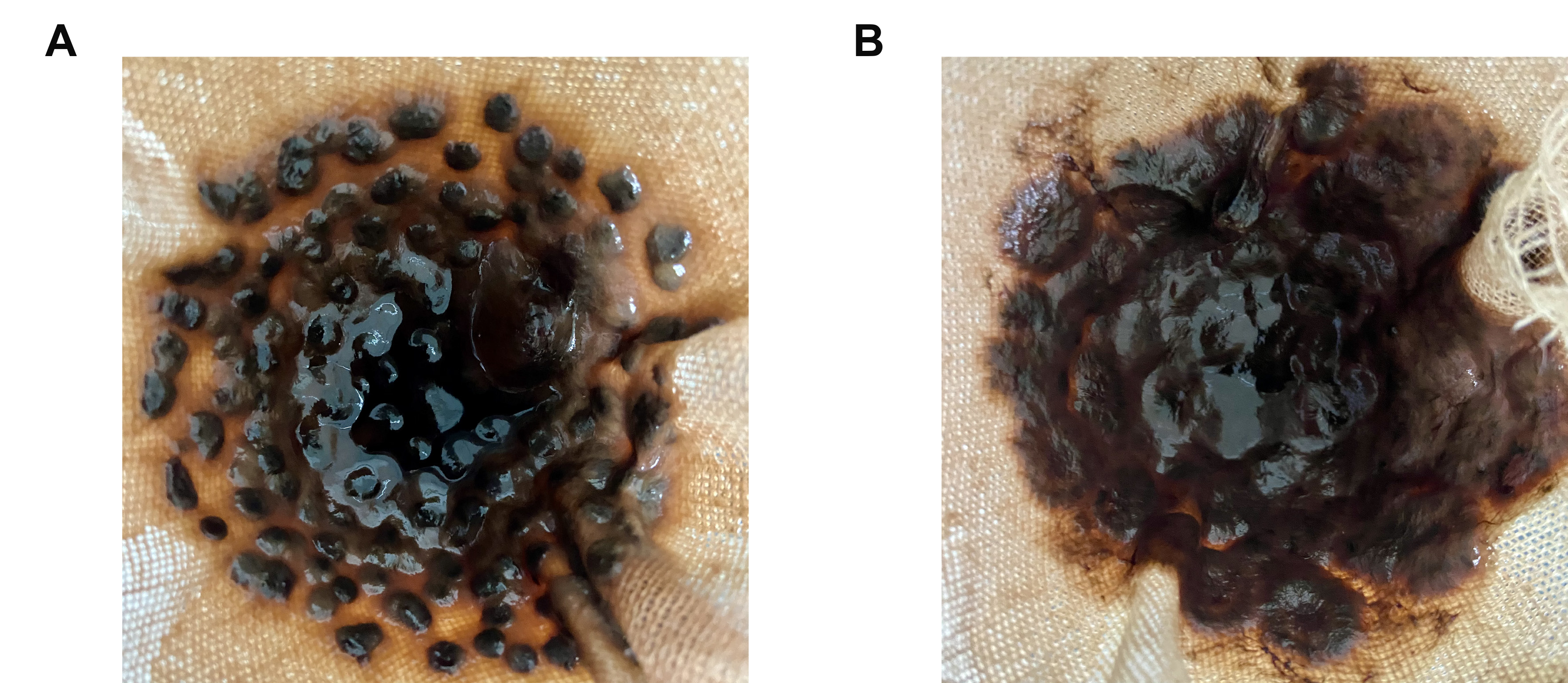


**Fig. S2** Autolysis of *Cercospora* sp. JNU001. **A.** The mycelia of *Cercospora* sp. JNU001 mycelia on day 11. **B.** The mycelia of *Cercospora* sp. JNU001 mycelia on day 13.


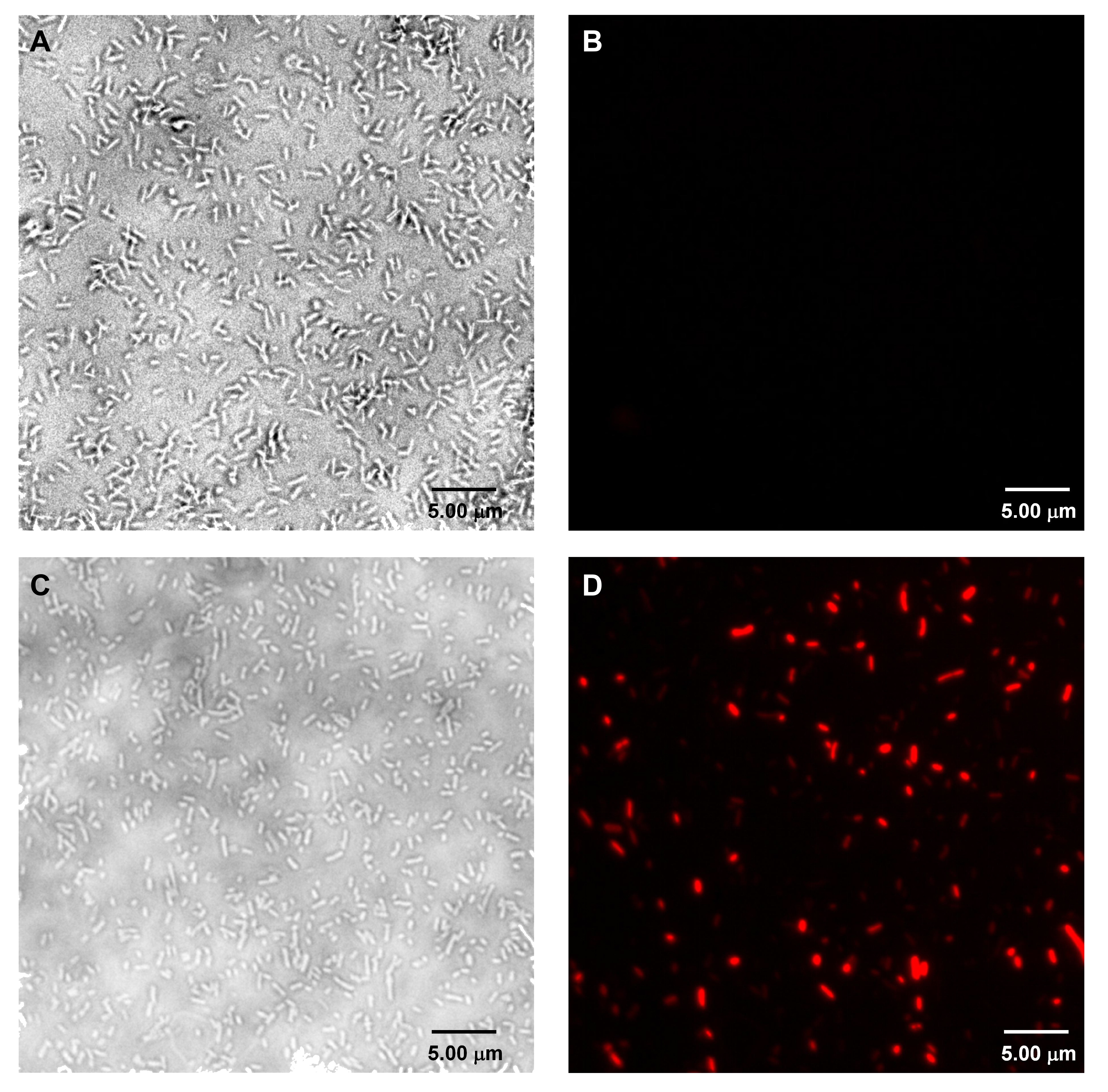


**Fig. S3** Fluorescence microscope observation of B15 after co-culturing with *Cercospora* sp. JNU001. **A.** The bright-field image (200×) of B15 alone. **B.** The fluorescence image (200×) of B15 alone. **C.** The bright-field image (200×) of B15 after co-culture with *Cercospora* sp. JNU001. **D.** The fluorescence image (200×) of B15 after co-culture with *Cercospora* sp. JNU001.


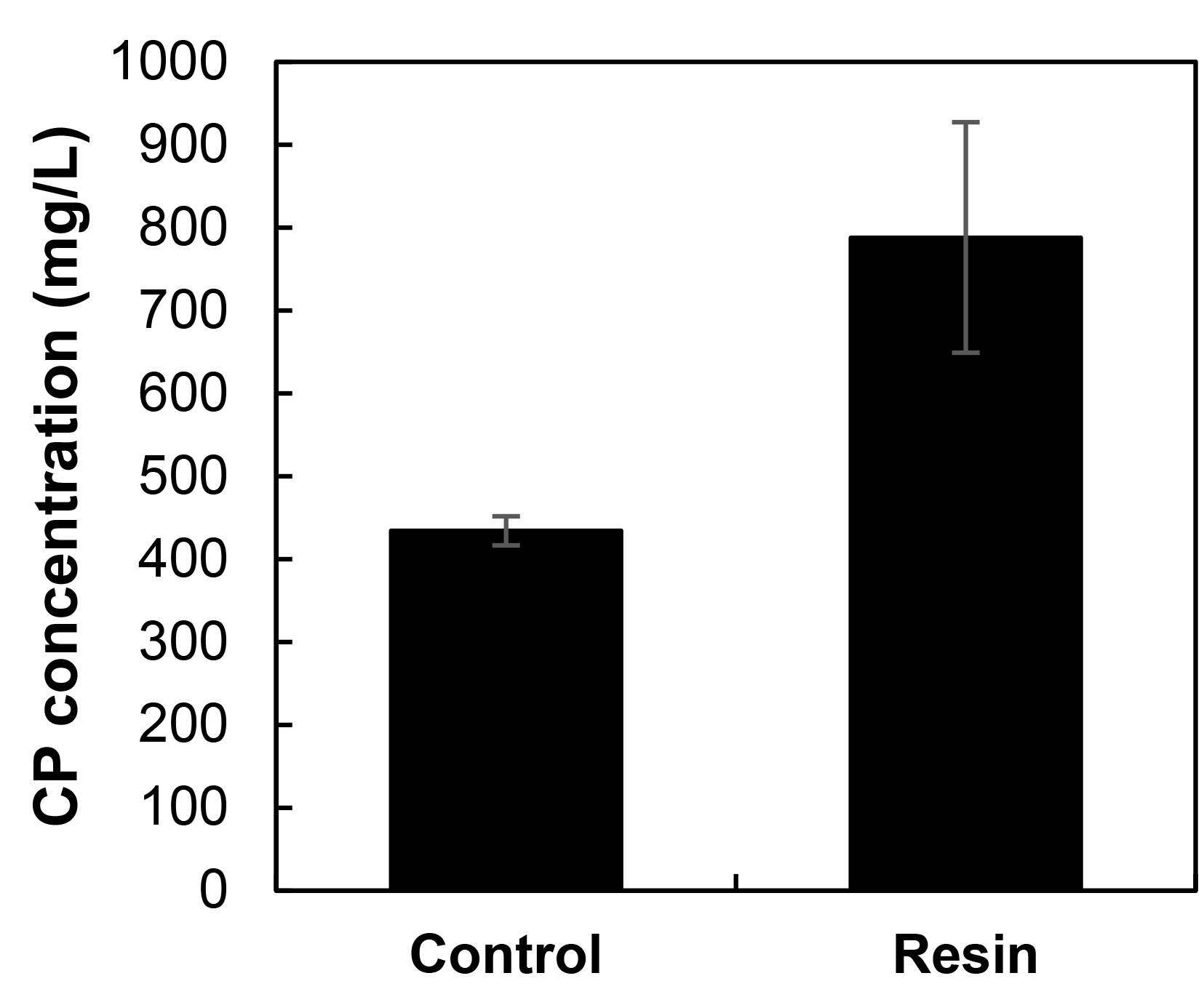


**Fig. S4** Effect of XAD-16 resins on the CP production. *Cercospora* sp. JNU001 cultured in modified S-7 medium as a control.


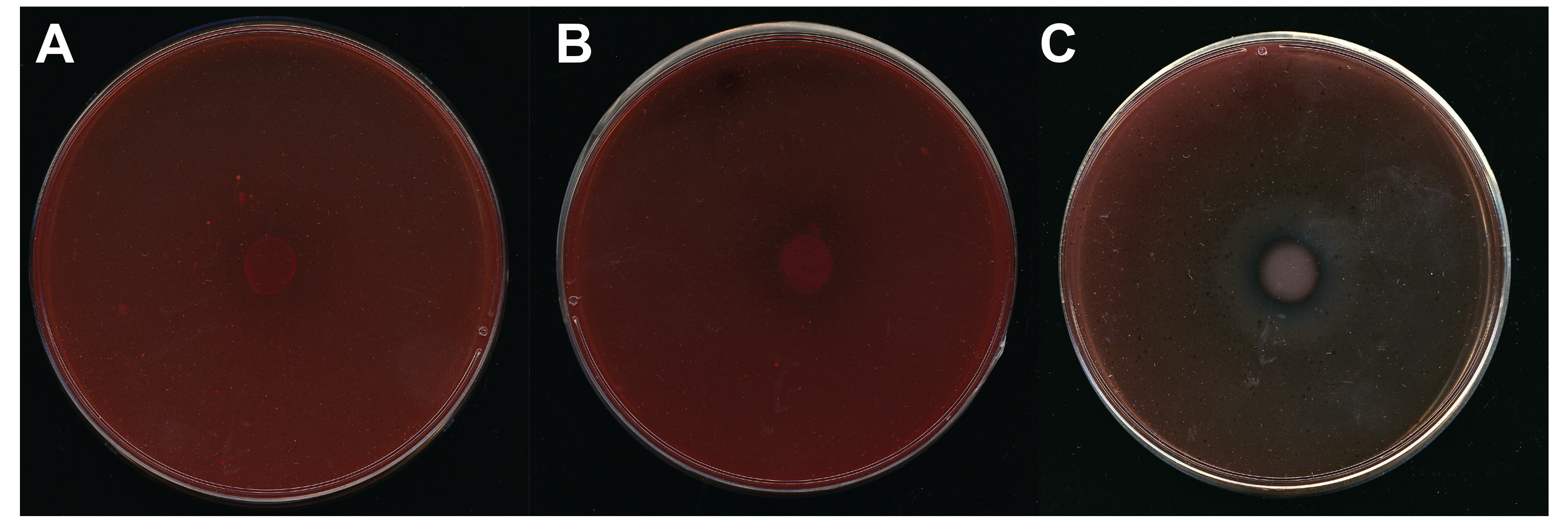


**Fig. S5** Analysis of the ability of B04 and B15 to degrade glucan. **A.** Glucan Congo red agar plate without bacteria. **B.** Glucan Congo red agar plate with B15. **C.** Glucan Congo red agar plate with B04.


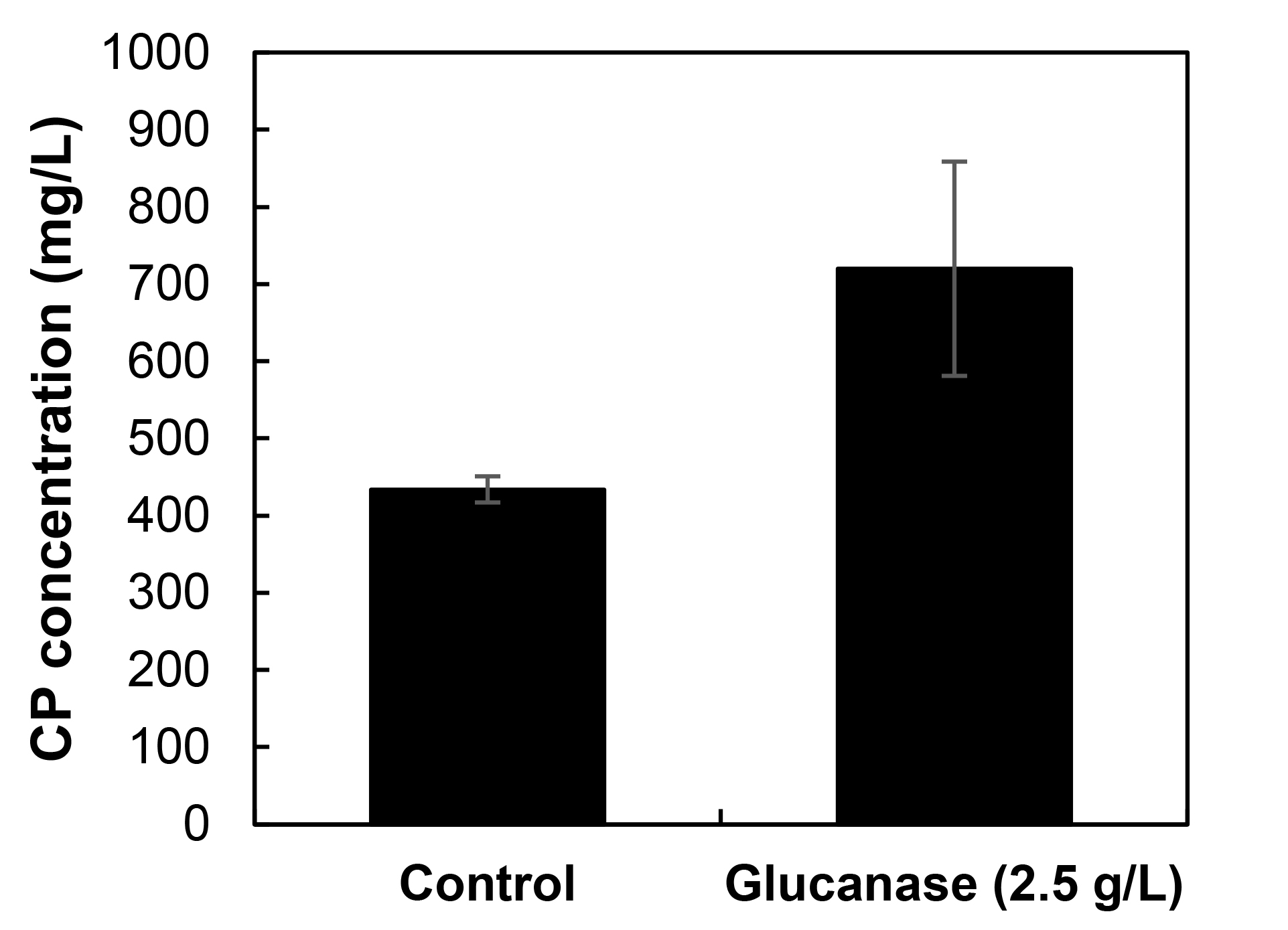


**Fig. S6** Influence of glucanase on the CP production. *Cercospora* sp. JNU001 cultured in modified S-7 medium as a control. Glucanase (50000 U/g) was purchased from Heshibi Biotech Ltd. (Yinchuan, China).


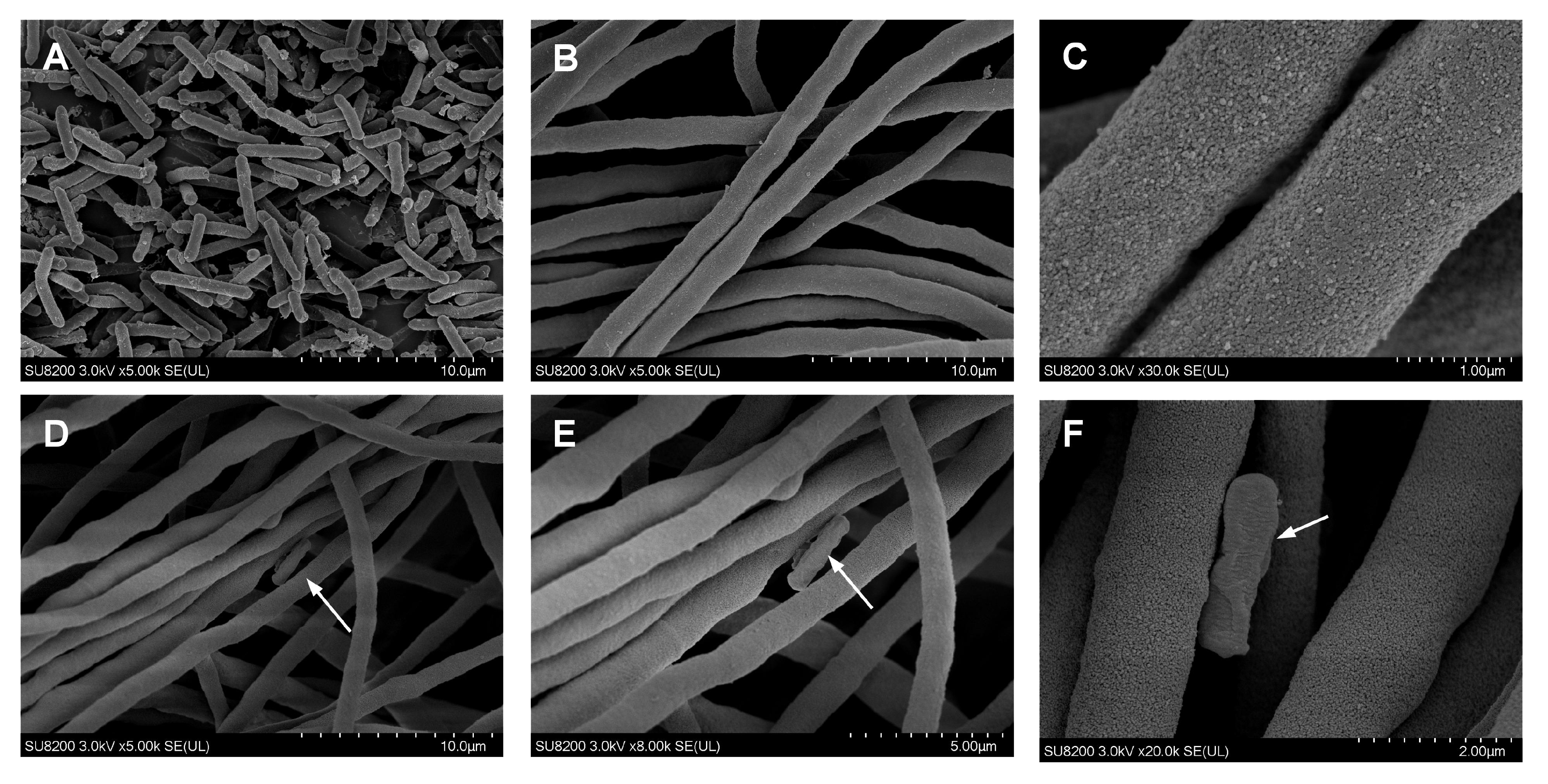


**Fig. S7** FESEM observation of *Cercospora* sp. JNU001 with B15. **A.** B15 samples. **B, C.** *Cercospora* sp. JNU001 samples. **D-F** Co-cultivation of B04 and *Cercospora* sp. JNU001. White arrows indicate bacteria B04. Scale bar was indicated.
